# Supplementary figures and images for: Characterization of Neisseria gonorrhoeae colonization of macrophages under distinct polarization states and nutrients environment
Source: Front Cell Infect Microbiol. 2024 May 14;14:1384611. doi: 10.3389/fcimb.2024.1384611 (PMC11130388; doi:10.3389/fcimb.2024.1384611)

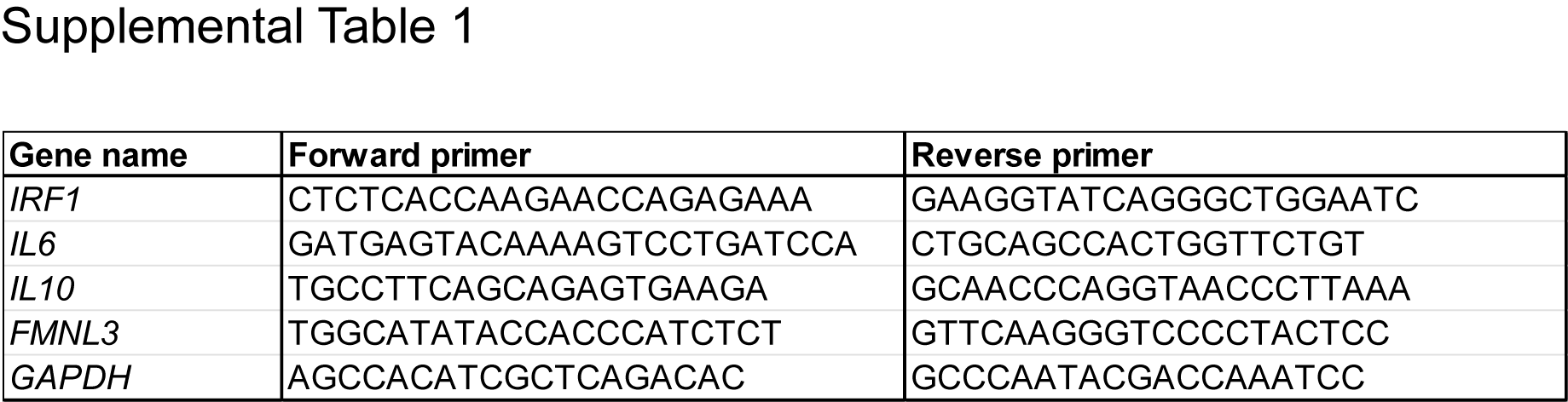

Supplement: Supplementary file 1 [file Image_1.tif]
